# Posterior intra-articular distraction with cage placement to treat congenital atlantoaxial dislocation associated with basilar invagination

**DOI:** 10.3171/2020.3.FocusVid.191001

**Published:** 2020-07-01

**Authors:** Wanru Duan, Dean Chou, Fengzeng Jian, Zan Chen

**Affiliations:** 1 Department of Neurosurgery, Xuanwu Hospital, Capital Medical University, Beijing, China; and; 2 Department of Neurological Surgery, University of California, San Francisco, California

**Keywords:** atlantoaxial dislocation, basilar invagination, irreducible, posterior approach, reduction

## Abstract

Congenital atlantoaxial dislocation (AAD) associated with basilar invagination (BI) is a complex congenital malalignment at the craniovertebral junction. The olisthesis, atlantoaxial facet joint arthropathy, and the contraction of the anterior soft tissue make the treatment challenging. Our video demonstrates the surgical technique for posterior intra-articular distraction with cage placement to treat congenital atlantoaxial dislocation associated with basilar invagination.

The video can be found here: https://youtu.be/7EQqW96HhN8

## Transcript

**0:20** This video demonstrates the surgical technique for posterior intra-articular distraction with cage placement in treating congenital atlantoaxial dislocation associated with basilar invagination (Duan et al., 2019; Goel, 2004).

**0:34** Clinical presentation. The patient is a 58-year-old woman who presented with progressive dizziness, weakness, and numbness of the right upper extremity for 2 years. The symptoms had worsened over the last 3 months. Neurological examination demonstrated unsteady gait, muscle strength grade 4 out of 5, and sensory disturbance of the right upper extremity.

**0:55** Imaging. A sagittal T2-weighted MRI shows the atlantoaxial dislocation and basilar invagination. The brainstem is compressed by the odontoid process, and there is associated syringomyelia in the upper cervical spine.

Three-dimensional CT reconstruction shows an assimilated C1, with fusion of the C1 lateral mass and the occipital condyle. There is also sagittal obliquity of atlantoaxial facet joints (Chandra et al., 2014; Salunke et al., 2015). The distance of the tip of odontoid above the Chamberlain’s line is 12 mm, and the atlantodental interval is 5 mm.

Hyperextension x-rays show that the atlantodental interval remained at more than 3 mm, defining this case as irreducible atlantoaxial dislocation.

CT angiogram shows a normal course of the vertebral artery.

This patient was treated with intra-articular distraction and cage placement through a single-stage posterior approach.

**1:57** Positioning. The patient was placed prone onto the operating table with cervical traction of one-eighth of the body weight (in this case 7.5 kg) (Wang et al., 2006).

**2:08** Exposure. A posterior midline incision was made, and subperiosteal dissection was performed from the external occipital protuberance to the C2 spinous process. The exposure was carried from bilateral facet joints to the transverse foramen of C2. Care was taken not to injure vertebral arteries.

**2:30** Releasing the facet joint. The atlantoaxial facet joints were already slightly distracted from the cervical traction. A microsurgical dissector was used to explore the intra-articular joints and the C2 pedicles.

The left intra-articular space was opened to 4 mm and a 4-mm trial was inserted into the space. A 4-mm intra-articular distractor was then inserted into the right facet joint and gently rotated until the intra-articular space was enlarged to 4 mm. The distractors were specially designed with blunt edges and enlarging sizes. A 4-mm trial was also placed into the right facet joint.

**3:13** After these had been done, the left-sided 4-mm trial was removed, and a 5-mm trial was placed into the right facet joint. A 5-mm intra-articular distractor was then used in the left joint and gently rotated until the intra-articular opening reached 5 mm. This process was continued, and the facet joints gradually enlarged with larger and larger distractors. Continued expansion and distraction was performed until the anterior soft tissue was released through this posterior approach (Duan et al., 2019).

**3:52** The sequential distraction continued until the facet joint was opened to a distance that was planned preoperatively (based upon the distance from the odontoid tip to Chamberlain’s line). The planned intra-articular opening in this case was 9 mm.

**4:10** An O-arm image showed that the basilar invagination had been completely reduced, and the atlantodental interval was significantly reduced.

**4:19** Intra-articular spacer implantation. The C2 nerve roots were mobilized cranially using a microsurgical dissector, and two interfacet spacers with height of 9 mm filled with autologous iliac crest bone graft were implanted into the bilateral intra-articular spaces. The angle of insertion of the spacers should be aligned with the surface of intra-articular joints in order to avoid undue force on the bony surfaces of the facets and possible fracture of the cortices.

**4:51** Fixation and cantilever technique. C2 pars screws (24 mm in length) were placed bilaterally.

An occipital plate with reduction set screws was attached to the occiput using midline screws. Preshaped rods were first placed into the C2 screw heads, and the C2 set screws were tightened, locking the distal end of the rods into C2.

Then the rods were reduced by cantilever into the occipital reduction screws, angling the C2 body forward and pushing the dens ventrally. Reduction of the atlantodental interval and basilar invagination was confirmed and the set screws were tightened.

**5:32** Postoperative imaging. A postoperative CT scan showed complete reduction of the atlantoaxial dislocation and of the basilar invagination. The clival-canal angle and cervical alignment were also improved. An MRI showed good decompression of the brainstem.

## Acknowledgments

Funding from the following is acknowledged: Beijing Municipal Natural Science Foundation (Beijing Natural Science Foundation Grant 7172091), Beijing Municipal Administration of Hospitals (Beijing Municipal Administration of Hospitals Grant PX2017002), and Beijing Municipal Health Commission (Beijing Health Commission Independent Innovation Fund 2018-2-2014).

## References

[1] Chandra PS Goyal N Chauhan A The severity of basilar invagination and atlantoaxial dislocation correlates with sagittal joint inclination, coronal joint inclination, and craniocervical tilt: a description of new indexes for the craniovertebral junction Neurosurgery 2014 10 suppl 4 621 630 25320950 10.1227/NEU.0000000000000470

[2] Duan W Chou D Jiang B Posterior revision surgery using an intraarticular distraction technique with cage grafting to treat atlantoaxial dislocation associated with basilar invagination J Neurosurg Spine 2019 31 4 525 533 10.3171/2019.4.SPINE1921 31277061

[3] Goel A Treatment of basilar invagination by atlantoaxial joint distraction and direct lateral mass fixation J Neurosurg Spine 2004 1 3 281 286 15478366 10.3171/spi.2004.1.3.0281

[4] Salunke P Sahoo SK Deepak AN Comprehensive drilling of the C1–2 facets to achieve direct posterior reduction in irreducible atlantoaxial dislocation J Neurosurg Spine 2015 23 3 294 302 26023897 10.3171/2014.12.SPINE14310

[5] Wang C Yan M Zhou HT Open reduction of irreducible atlantoaxial dislocation by transoral anterior atlantoaxial release and posterior internal fixation Spine (Phila Pa 1976) 2006 31 11 E306 E313 16688020 10.1097/01.brs.0000217686.80327.e4

